# Promotion of Officers

**Published:** 1918-02-09

**Authors:** 


### TERRITORIAL MEDICAL CORPS.

### Promotion of Officers.

The first report of the Committee on the promotion of officers in the Special Reserve, New Armies, and Territorial Force, was issued in July 1917. A second report, which deals with the promotion and rates of pay of officers of the Special Reserve and Territorial Force sections of the Royal Army Medical Corps, was sent in on September 1 last. It is now publicly announced that the Government has approved and are taking action on the recommendations of Col. Lord Burnham's Committee, with the exception of the first, proposing that officers of the R.A.M.C. Territorial Force and Special Reserve, who joined before the war, should be put on a level with temporarily commissioned contract officers as regards pay, allowances and gratuities, when they would gain by the change. The September report deals with certain grievances and anomalies, in regard to which Colonel Lord Burnham's Committee make recommendations which will remove them. The report as it stands is necessarily technical, and with a view to making the position clearer to those interested we sent it for the comments of a correspondent, who is a master of the technicalities, and thoroughly familiar with the points raised and the questions involved. He writes:

The British Empire refused to prepare itself for a great war till the great war came. Then preparation and training had to be hasty improvisation. Hence anomalies. War always must bring hardship to many, loss of money, loss of business—not to speak of disablement and death—when war means a nation struggling for its life. The medical arrangements ready for a war were the Royal Army Medical Corps Regular Service, Special Reserve, and Territorial. Of these the first had contracted to devote their days to the medical service of the Army, in peace or in war, at a certain rate of pay and allowance. They have no grievance, and do not complain. The lower ranks noted and commented on the fact that the temporarily commissioned officer gets a greater pay than they, but do not consider it a grievance. In fact, since all their service they are earning a pension worth about £4,000, they have about the same emoluments. The Special Reserve officer and the Territorial officer of the Medical Service also made a certain contract, and most patriotically had set themselves to prepare to do their part of every citizen's duty—that is, to defend their community from foreign foes. They made sacrifices. The patriotism that must be paid for in full would not be sacrifice. These men came to the Colours, and have done magnificent work. But the need of the Army was greater than these three classes of medical men could meet, and so temporary commissions at a certain rate of pay were offered to those, who had not prepared themselves before the war was on us. The pay was higher than the pay agreed on for the Special Reserve and the Territorial officers. In fact, those who came at the eleventh hour got not only the same, but more than those who came first. Nineteen hundred years ago that question was discussed. There was an anomaly, not a grievance.

It is probable that it would be the better plan to treat all Territorial medical officers as the R.A.M.C. Regular Army is treated, and to put them on one list. The trouble would come in time of peace, for it might happen that in one district all the officers were senior, in another all were junior, and that distribution could not be made. But in war-time it would work fairly. The Territorials may have made a mistake in keeping themselves in Territorial units. A more elastic system might be better. Apparently a general list is to be set forth.

Regarding administrative appointments it must be remembered that twenty-years' commissioned service, outside the regular R.A.M.C., is not the same as twenty years' service in it. It would be a grievance to the Regular Service if it were often the case, that, the senior men were kept out of administrative appointments in war-time by men who showed a year or so more of commissioned service than they, but whose commissioned service, in many of those years only, was a few weeks' work. Moreover, it is certainly the case, that the Regular officer selected for an administrative appointment is usually better, than the Territorial or Special Reserve officer selected for such appointment. As medical men there is nothing to choose between the average Regular, the average Special Reserve, and the average Territorial. As administrative officers, the men of the Regular Service marked for the average Regular, the average Special Reserve, or Territorial, usually, probably because they have so long been used to be commanded and to obey orders, the Regular is a better commanding officer than the Special Reserve or Territorial officer. After all, this is only what should be expected.

A Regular medical officer in an administrative billet has expressed to us his opinion that the medical profession, Special Reserve, Territorial, and temporarily commissioned men alike, have come out splendidly during this war, and has told us that he himself never considers what branch a man belongs to when he selects him for a job, and often does not know. "It is the rarest thing in the world for a man to fail me. I have been splendidly served." Such are his words, and we can vouch he is a man who would not say them if he did not believe them. As the result of Colonel Lord Burnham's committees, the anomalies seem likely to disappear. When peace dawns over the land, the medical profession will be able to return to the ways of peace feeling proud of the work it has done, and the courage and sacrifice of its members. We are sure the Regular Service will gratefully acknowledge the good comradeship and assistance it has received from its civilian brothers.

